# The Effects of Assisted Reproductive Technologies on De Novo Mutations

**DOI:** 10.32607/actanaturae.27589

**Published:** 2025

**Authors:** N. A. Arakelyan, J. Vasilevska, E. I. Rogaev

**Affiliations:** Center for Genetics and Life Science, Sirius University of Science and Technology, Sirius, 354340 Russia; Department of Psychiatry, UMass Chan Medical School, Worcester, MA 01545 USA †Equal contribution

**Keywords:** assisted reproductive technologies, de novo mutations, aging, paternal age effect, maternal age effect

## Abstract

Recent advances in assisted reproductive technologies (ART) have revolutionized human reproduction, offering hope to millions of couples facing infertility issues. At the same time, concerns persist regarding the potential impact of ART on the genomic integrity of offspring conceived through these techniques. Specifically, questions abound about the effects of these techniques on the incidence of de novo mutations (DNMs), which are genetic alterations that arise spontaneously in the germline or during early embryonic development and are implicated in various human diseases. The extent to which ART directly affects the rate of de novo mutations has been the subject of ongoing debate. This review explores recent studies that have investigated the relationship between ART and DNMs. It underscores the necessity for further research to clarify the clinical implications and long-term consequences of ART.

## INTRODUCTION

Since the birth of the first child conceived through in vitro fertilization more than 45 years ago, the technology has become commonplace in reproductive medicine. The prevalence of couples experiencing infertility has been on a consistent upward trend in recent decades, particularly as an increasing number of individuals postpone their intentions to conceive until a later age. A significant number of these couples ultimately turn to artificial reproductive means. Latest estimates indicate that over 10 million infants have been born globally as a result of assisted reproductive technologies (ART), and that approximately 4 million ART cycles are recorded every year [1, 2].

ART refers to fertility treatments that include all interventions related to the in vitro handling of both human oocytes and sperm or embryos for reproduction [3]. Gametes and zygotes undergo a range of non-physiological processes and are exposed to culture media during ART treatments. While the majority of infants conceived through ART are born healthy, concerns persist regarding the safety of these technologies and the potential long-term effects of ART on the development, growth, and overall health of this emerging population [4].

*De novo* mutations (DNMs), which arise spontaneously in the germline or during early embryonic development, have the potential to shape the genetic outcomes of offspring conceived through ART. A comprehensive understanding of the mechanisms underlying the occurrence of de novo mutations and their origin is crucial for addressing the safety and efficacy of ART. Such insights facilitate the development of strategies aimed at mitigating risks and improving ART procedures, with the objective to minimize potential adverse effects on the genetic integrity and health of subsequent generations. The investigation into the relationship between ART and DNMs not only deepens our understanding of the underlying molecular mechanisms involved but also highlights the importance of making informed decisions regarding reproductive interventions [5, 6].

This review seeks to synthesize recent evidence concerning the association between ART and DNMs, given the extensive application of ART and its potential implications for genetic integrity. The objective is to inform clinical practice and future research in the field of reproductive genetics.

## ASSISTED REPRODUCTIVE TECHNOLOGIES (ART)

Assisted reproductive technologies comprise a wide range of procedures aimed at addressing infertility in both females and males. They have witnessed a significant increase in utilization over the years. The approach entails a series of complex steps, including ovarian stimulation, oocyte and sperm preparation, gametes and embryos manipulation, cryopreservation, in vitro culture, and, ultimately, embryo transfer. The extent of the intervention can vary considerably, ranging from hormonal treatments for patients to more sophisticated in vitro procedures. These interventions encompass a spectrum of techniques, beginning with subtle manipulations of gametes, such as in vitro fertilization (IVF), and extending to more invasive procedures, including intracytoplasmic sperm injection (ICSI) and testicular sperm extraction, in conjunction with intracytoplasmic sperm injection (TESE-ICSI). Furthermore, these interventions also involve direct alterations to the embryo, as illustrated by procedures such as trophectoderm or blastomere biopsy (BB). These pivotal stages align with essential developmental phases, characterized by substantial epigenetic remodeling of the genome and increased susceptibility to environmental factors. Any disruption during these critical moments has the potential to adversely impact developmental programming [7, 8]. One key factor contributing to the increased risk associated with ICSI is the sperm selection process. In contrast to conventional IVF, where sperm must independently penetrate the protective layers of the egg, ICSI circumvents these natural selection mechanisms by manually selecting and injecting a single sperm directly into the egg. This process may unintentionally introduce sperm that carry DNA anomalies into the fertilization process. Moreover, the ICSI often involves the retrieval of sperm directly from the testes or epididymis, thereby bypassing the natural maturation process that typically occurs during the sperm transit through the male reproductive tract. The likelihood of genetic abnormalities appearing in offspring may increase as a result of this procedure. Furthermore, the invasive nature of ICSI may elevate the risk of causing damage to the oocyte or the meiotic spindle apparatus during the injection process. Such damage could disrupt DNA replication and repair mechanisms, thereby potentially affecting embryo development [9, 10]. Although the majority of children born through ART are healthy, numerous studies have identified associations between ART and various adverse health outcomes [11, 12, 13, 14, 15, 16]. These include congenital heart defects [17] and childhood cancers [18]. However, inconsistencies persist in the literature concerning the increased risks of impaired cognitive development, neurodevelopmental disorders [19], metabolic health, and the effect on reproductive fitness associated with ART [20, 21, 22]. Despite the existence of these health risks, it remains unclear whether they are attributable to specific ART procedures, such as sperm retrieval, ovarian stimulation, methods of conception, or laboratory conditions, or whether they are influenced by underlying parental factors or exacerbated by the circumvention of natural selection. Moreover, the interplay between epigenetic and genetic factors in relation to these health outcomes is not yet fully understood. While research on epigenetics within the context of ART is expanding, the investigation of DNMs remains in the nascent stages of exploration. Despite considerable advancements in our understanding of epigenetics, research continues to comprehensively elucidate the role of de novo mutations in the context of ART-conceived offspring [23, 24, 25, 26].

## DE NOVO MUTATIONS

Human de novo mutations (DNMs) are defined as the development of new genetic alterations that appear in the germline during one generation. Germline de novo mutations (gDNMs) occur in the gametes of one parent and are transmitted directly to the offspring at the time of conception. In contrast, post-zygotic de novo mutations occur during the early embryonic development and can affect a significant proportion of the offspring’s cells. The implications of a new genetic mutation can vary considerably. While neutral or beneficial mutations may become integrated into the genome of our species, thereby contributing to human evolution, alterations in critical genetic sequences can also disrupt biological systems, potentially resulting in severe diseases. De novo mutations are the most severe type of uncommon genetic variations, often more harmful than inherited variations due to less stringent evolutionary selection [5, 27]. They have been linked to various diseases [28], including neurodevelopmental disorders [29, 30, 31], heart diseases [32], early-onset high myopia [33], and may also play a role in cases of rare sporadic malformation syndromes like Schinzel– Giedion, Kabuki, and Bohring–Opitz syndromes [34]. Recent studies also indicate their fundamental effects in male and female infertility [35, 36].

De novo mutations can occur at various stages of development, including prior to fertilization in the germ cells, as well as during the cleavage and blastocyst stages of early embryonic development. DNMs encompass a range of mutation types, like point mutations or single-nucleotide variants (SNVs), insertions and deletions (indels) of varying sizes, as well as larger copy number variants (CNVs) and structural variations. Typically, DNMs are identified through the analysis of DNA samples obtained from trios consisting of a father, mother, and offspring. On average, a human genome is estimated to contain between 44 and 82 de novo single nucleotide variations, with 1 to 2 of these occurring in coding regions [37, 38, 39]. Furthermore, the distribution of DNMs is not random across the genome. Instead, specific genomic features—such as the guanine-cytosine content, nucleosome occupancy, repeat content, recombination rate, transcriptional activity, replication timing, and chromatin state---significantly affect the likelihood of mutations occurring in certain regions. Multiple DNMs may occur in close proximity within the same individual’s genome, a phenomenon referred to as mutational clusters. In contrast, mutational hotspots are characterized by the occurrence of multiple DNMs at the same location across several individuals. It has been demonstrated that the rate of genetic mutations in the human germline, encompassing the entire genome, is affected by the specific class of genetic variants in question [40]. The prevalence of germline DNMs in humans significantly varies both within and among families, with these variations influenced by sex. The rate of germline DNMs is believed to be potentially modulated by intrinsic, as well as extrinsic, factors. One of the most important findings concerning germline DNMs is the observation that their frequency increases progressively with the age of both parents at the time of conception, particularly with paternal age. Specifically, there is an increase of approximately 1.35 to 1.5 DNMs in the offspring’s germline for each additional year of paternal age. In contrast, the increase in DNMs associated with advancing maternal age is considerably less pronounced, with an addition of only 0.24 to 0.42 DNMs for each additional year of maternal age [41, 42, 43, 44, 45, 46, 47]. Approximately 2–3% of all DNMs in the offspring occur in proximity to one another (below 20 kb), forming what are referred to as clustered DNMs (cDNMs). These clusters exhibit a greater maternal bias compared to paternal clusters, which can be attributed to the deficient homologous recombination repair of double-strand breaks associated with the DNA repair mechanisms of aging oocytes. This impairment facilitates the emergence of deregulated recombination hotspots, resulting in mutations that occur in closer proximity than would typically be anticipated. It is noteworthy that 58.4% of maternal cDNMs are enriched within specific genomic regions on the chromosomes 8, 9, and 16, although these regions are also characterized by a higher frequency of maternal unclustered DNMs. In contrast, the frequency of paternal cDNMs correlates with chromosome length. This disparity is linked to the distinct mechanisms that underlie the etiology of de novo mutations in different sexes [5, 45].

The predominant hypothesis behind the paternal age effect suggests that mutations arise as a result of random errors during genomic replication. This replication predominantly occurs within the male germ line to facilitate the ongoing production of sperm. Spermatogonia, which serve as the stem cells for spermatogenesis, undergo a series of divisions to both self-renew and produce spermatocytes, which subsequently differentiate into sperm. As males advance in age, the frequency of divisions per spermatogonium rises, thereby increasing the likelihood of new mutations arising from replication errors. Consequently, mutations accumulate in spermatogonia as age advances, thereby illustrating the paternal age effect. While this "selfish selection" model provides insight into the origin of dominant developmental disorders like achondroplasia and Apert syndrome, it fails to extend to the majority of DNMs that do not exhibit an apparent selective bias. Alternatively, recent research has proposed a hypothesis that challenges the traditional perspective, which posits that replication errors are the primary source of human germline mutations. This new hypothesis suggests that the consistently low rate of spontaneous mutations attributed to replication errors associated with paternal age may be augmented by mutations resulting from defective DNA repair mechanisms in the male germ line [5, 37, 48, 49].

The mechanisms underlying the maternal age effect cannot be attributed to genome replication, as oocyte production ceases during the intrauterine phase, and genome replication is no longer active. Therefore, the mechanisms underlying age-related DNMs in mothers must fundamentally differ from those associated with paternal aging. Evidence suggests that maternal age at conception affects the mutation frequency, which may be the result of accumulated damage in the oocytes or potentially materialize via post-zygotic mutations occurring in the embryo. Following fertilization, the oocyte has a limited timeframe to repair DNA damage introduced by the spermatozoon prior to the activation of the S-phase in the first mitotic division. Faulty or deficient repair during this process may lead to mutations that impact the health of offspring, a phenomenon referred to as the "post-meiotic oocyte collusion hypothesis" [50, 51, 52]. Such a mechanism aligns with recent data indicating that (ART) medical procedures may increase the mutational burden carried by offspring conceived through these procedures [52].

**De novo** **mutations associated with ART**

Numerous studies have investigated natural conception and the prevalence of DNMs [53, 54, 55]. However, there is a paucity of research examining the relationship between conception through ART and DNMs (Table 1). Although many studies generally indicate that ART is safe [56, 57, 58, 59], recent evidence suggests that ART may be associated with an increased burden of DNMs in children conceived via this method [6, 20, 35, 60, 61].

**Table 1:** Investigations into ART and de novo mutations

Case group (n)^*^	Control group (n)	Relevant outcomes of the study	Study
12 ICSI	30 SP	In six out of the twelve children who had undergone ICSI, ten apparent de novo “genomic copy number changes in one direction” were identified, characterized by simultaneous gains or losses in the copy number relative to both biological parents, with a particular emphasis on losses.	[20]
25 trios ARTP	693 trios SP	The application of ART had a moderate significance (P = 3.86 × 10−3), with an average increase of 4.25 DNMs compared to natural conception after controlling for other variables.	[56]
49 quarters2 IVF	62 quarters2 SP	De novo numerical aberrations or large structural DNA imbalances occur at comparable frequencies in IVF and those conceived naturally.	[57]
2505 ICSI: 1114 fetuses prenatal tests, 1391 fetuses postnatal tests	–	The incidence of de novo chromosomal abnormalities in the pre-and postnatal karyotypes of ICSI offspring was found to be higher compared to the general population, and this increase was associated with the sperm parameters of the fathers.	[60]
116 trios, 86 quartets (twin offspring) ARTP	205 trios SP	Children conceived via ART exhibited an average of 4.59 more gDNMs compared to those conceived spontaneously. This difference includes 3.32 paternal and 1.26 maternal DNMs, after adjusting for factors such as parental age at conception, cigarette smoking, alcohol consumption, and exercise load.	[6]
49 trios and 2 quartets ARTP (17 IVF, 18 ICSI-TESE+ICSI-TESE)	18 trios SP	No significant differences were observed in the number of DNMs per child across various methods of conception, regardless of the parental age at the time of conception.	[58]
1496 ARTP (IVF/ICSI)	1396 SP	The IVF/ICSI group exhibited a slight increase in the overall rate of de novo chromosomal abnormalities compared to the NC group; however, this difference did not reach a statistical significance.	[62]
535 ARTP with suspected genetic conditions	1316 SP with suspected genetic conditions	The proportions of de novo variants observed in the ART group were comparable to those in the non-ART group.	[59]
270 ARTP, 67 ARTP mother–child pairs, 113 oocytes were donated in natural menstrual cycles	181 SC, 90 SC mother–child pairs, 113 oocytes donated after OS	ART individuals have a higher prevalence of de novo non-synonymous mtDNA variants compared to their counterparts conceived spontaneously.	[61]

Trios – mother, father, and child.

Quartets^1^ – mother, father, and two siblings.

Quartets^2^ – father, mother, placenta, and neonate umbilical cord blood.

ARTP – ART pregnancies (IVF + ICSI).

SP – spontaneous pregnancies (natural cohort).

IVF – in vitro fertilization.

ICSI – intracytoplasmic sperm injection.

TESE-ICSI – testicular sperm extraction associated with intracytoplasmic sperm injection.

^*^The participants were not diagnosed with any recognized genetic disorders unless so indicated.

In their pivotal study, Wang et al. investigated the potential link between ART and the incidence of congenital heart defects (CHDs) in offspring, with a specific emphasis on DNMs as a contributing factor. The researchers conducted whole-genome sequencing on a cohort comprising 1,137 individuals from 160 families who conceived spontaneously and 205 families who conceived through ART. The findings indicated that children conceived via ART exhibited a significantly higher frequency of germline DNMs (gDNMs) compared to those conceived spontaneously, even after adjusting for various confounding factors such as parental age, smoking habits, alcohol consumption, and levels of physical activity. In particular, offspring conceived through ART exhibited a 4.59-fold increase in germline de novo mutations (gDNMs), with 3.32 mutations originating from paternal sources and 1.26 from maternal sources [6]. This finding aligns with a study by Wong et al., which reported that the application of ART for conception resulted in an average of 4.25 additional DNMs per genome compared to natural conception [56]. Notably, paternal DNMs in ART-conceived offspring were characterized by specific C>T substitutions at CpG sites, which have been associated with an elevated risk of CAD. Furthermore, the study indicated that a significant majority (87.9%) of these mutations were inherited from the father, while the utilization of both recombinant and follicle-stimulating hormone, along with a high-dose human chorionic gonadotropin trigger, was correlated with an increase in maternal DNMs [6]. A contrasting study did not observe any significant differences when directly comparing the mitochondrial DNA (mtDNA) variants in oocytes obtained through natural means and those retrieved following ovarian stimulation cycles. Nevertheless, this study identified a correlation between the presence of non-synonymous mtDNA heteroplasmic variants in protein-coding regions and ribosomal RNA loci and a reduction in birth weight. These variants were found to be prevalent in children born to older mothers who had undergone ART treatments. It is hypothesized that these variants may induce a mild, yet significant mitochondrial dysfunction, which could contribute to a lower birth weight percentile. This finding provides preliminary evidence of mitochondrial genetic factors that may help explain the disparities in condition between individuals conceived through ART and those conceived naturally [61].

As previously noted, ICSI has generated greater concerns compared to other ART procedures due to its invasive nature and the potential risks associated with the selection and manipulation of sperm. However, it is important to acknowledge that there exists contradictory data on this matter [23, 63, 64]. In this context, several research groups have studied the specific effect of ICSI on DNMs. A small study conducted by Woldringh et al. indicated a relative abundance of ‘same direction copy number changes’ in an ICSI setting, with an increase by a factor of five compared to the control population. Nevertheless, due to the limited size of the study group, further research with a larger cohort is necessary to validate these findings [20]. Significantly higher rates of de novo chromosomal abnormalities have been observed in ICSI-mediated offspring. These abnormalities include sex chromosome aneuploidies and structural chromosome anomalies, particularly reciprocal translocations [65]. The incidence of de novo fetal karyotype abnormalities was determined to be 3.2%, primarily attributed to a substantial number of de novo autosomal abnormalities rather than sex chromosome abnormalities. Furthermore, the incidence of de novo chromosomal abnormalities, reported at 1.9% in a pooled sample of ICSI offspring assessed both pre- and postnatally, is notably higher compared to the general population and is associated with the paternal sperm count. Higher rates of de novo chromosomal abnormalities have been found in the fetuses and children of couples in which men exhibited below-reference sperm concentration and total sperm count. It is important to note that the frequency of abnormal de novo prenatal test results did not demonstrate a statistically significant difference when comparing the use of ejaculated versus non-ejaculated sperm or between fresh and frozen-thawed sperm. However, the absence of a control group in the study may have had an impact on the observation of a higher prevalence of karyotype abnormalities. Additionally, it is widely recognized that pregnancies achieved through ART undergo more comprehensive prenatal karyotype screening compared to those conceived naturally. Consequently, to investigate whether the higher incidence of karyotype anomalies following ICSI is a result of closer attention, the authors suggest that the optimal control group should consist of spontaneously conceived pregnancies that adhere to similar screening practices, thereby facilitating a more rigorous analysis [60].

Conversely, other research has not identified a correlation between the number of DNMs and ART. Hunag et al. conducted an investigation into the type of molecular defects present in neonates conceived via ART who were admitted to the neonatal intensive care unit with suspected genetic conditions. The genetic profiles of these neonates were compared to those of neonates conceived without ART. The study found no significant differences in the genetic profiles between the two groups, which encompassed the rates of diagnosis and the proportion of de novo variants (DNVs), as well as the percentage of SNVs and CNVs between the two cohorts. That study presents several limitations. As noted by the authors, the research focused exclusively on live-born children, thereby excluding stillborn fetuses and pregnancies that had been terminated. This omission may affect the assessment of certain fetal lethal SNVs or CNVs. Additionally, well-documented potential risk factors, such as advanced paternal age, unhealthy parental lifestyles, and infertility, were not examined [59]. The findings align with those by Smits et al., who revealed no significant differences in the number of DNVs between 53 children and their parents [58]. However, the study conducted by Smits and colleagues did not explore the impact of ART on the prevalence of somatic DNMs in the offspring or the occurrence of CNVs. Esteki et al. investigated the prevalence and extent of de novo large CNVs greater than 100 kb in live-born neonates conceived through IVF compared to those conceived naturally. The researchers profiled the genomic landscape of fetal and placental tissues postpartum to assess the prevalence and persistence of large genetic aberrations that may be attributed to the chromosomal instability (CIN) associated with IVF. The findings revealed that approximately 10.8% of pregnancies exhibited sporadic de novo genomic aberrations distributed throughout the genome, indicating that these events are random and infrequent occurrences of embryonic CIN, with no discernible functional implications for placental biology or fetal health [57]. It is important to highlight that ART is used to mitigate the risk of pathogenic de novo mutations, which are more prevalent due to increased mosaicism in parents. This condition elevates the likelihood of recurrence, thereby increasing the risk that a subsequent child may be affected by the same DNM as the preceding one [66].

While our primary focus has been on ART procedures on humans, it is important to acknowledge that research conducted on animal models has also yielded conflicting results. For example, studies involving transgenic mice have demonstrated no significant difference in the frequency or spectrum of DNMs between naturally conceived fetuses and those produced through various ART techniques [67]. Nevertheless, there exist additional data points to consider. The genealogy of domestic cattle is meticulously documented across numerous generations and is characterized by extensive paternal and maternal half-sibling pedigrees. This comprehensive record-keeping presents unique opportunities to construct large pedigrees that are particularly well-suited to the identification of DNMs. Furthermore, the reproduction of domestic cattle extensively employs ART, which includes methods such as artificial insemination (utilizing frozen semen for female insemination), IVF, and ICSI. These techniques facilitate the examination of the impact of ART on DNMs.

**Fig. 1:**
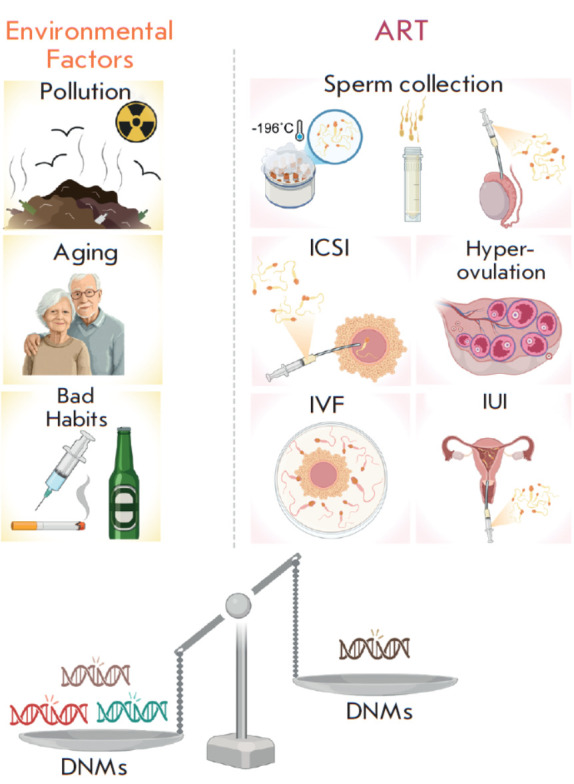
Factors contributing to de novo mutations (DNMs). Two categories of factors that influence the accumulation of DNMs are illustrated: environmental factors (left panel) and assisted reproductive technologies (ART) (right panel)

When examining species such as cattle, which are particularly suited for investigating this phenomenon, it becomes evident that the correlation between DNMs and the use of ART is significant. Notably, IVF has been shown to increase the rate of de novo structural variation (dnSV) by approximately fivefold, with the associated mutations occurring during the very early stages of embryonic development, specifically during the one- and two-cell stages [68]. While ART procedures contribute to the appearance of DNMs, their impact has been shown to be less significant compared to parental aging and other environmental factors (summarized in Fig. 1). In summary, while the research conducted in this field has several limitations, the findings related to cattle suggest that further investigations are necessary.

## AGE AS A LIMITING FACTOR OF ART

One of the most contentious issues within this context is the consideration of parental age as a basis for restricting access to ART. For instance, in their publication, Bewley et al. emphasized the importance of providing women with comprehensive information regarding reproduction and fertility, as well as the limitations of ART as a means to achieving pregnancy at an advanced age. While their discourse primarily focuses on the impact of biological factors on fertility and the prospects for a healthy pregnancy, they ultimately convey the notion that there exists a specific chronological timeframe during which childbearing is most advisable. Indeed, their subtitle asserts that the most secure age for childbearing remains 20–35 years, against the potential risks associated with ART for women exceeding this age range [69].

The age restrictions on the application of ART vary among countries. For instance, in the United States, a notable exception to the general absence of age restrictions in statutory law is reflected in the guidelines issued by the Ethics Committee of the American Society for Reproductive Medicine. This committee has explicitly recommended against providing donor oocytes or embryos to women over the age of 55, even in the absence of any underlying medical conditions. The authors justify the establishment of the age limit of 55 by citing the limited data available regarding maternal and fetal safety, as well as concerns related to longevity and the necessity for sufficient psychosocial support for raising a child to adulthood.

The legislation regulating ART throughout Europe reveals that most of the countries have established a chronological age limit for potential mothers, typically ranging from 45 to 50 years. For instance, the Czech Republic has set the age limit for women seeking access to ART at 49 years [70]. In Belgium, prospective mothers must be under 45 years of age to request access to ART and the implantation of an embryo or insemination is not permitted after the age of 48 years [71]. A report from the Swedish National Council on Medical Ethics indicates that there are no mandated upper age limits for IVF in Sweden. However, it is noted that county councils typically impose upper age limits ranging from 37 to 41 years. The report asserts that at least one parent must be of a sufficiently young age to assume responsibility for the child until the child reaches adulthood [72]. This assertion aligns with the Swiss position, which stipulates that both prospective parents should be of an age that enables them to adequately care for their child until the child reaches the age of majority [71]. Estonia, Greece, and the Netherlands set the maternal age limit at 50 years [70]. Notably, a social study investigating public perceptions regarding maternal age revealed that 43 percent of participants believed that women should stop bearing children as they reach the age of 50 [73].

It is noteworthy that, despite the increased risks of health issues in offspring closely associated with advanced paternal age, fathers generally face fewer restrictions regarding the application of ART. In Sweden, prospective fathers are not permitted to be older than 56 years, while in Finland and Portugal, the age limit is set at 60 years [71]. A recent social study performed in the United States revealed that the preferred upper age limit for men was 64 years [74].

Based on an analysis of scientific studies and legislative approaches across various countries, it can be concluded that parental age is a significant factor influencing access to ART, particularly for women. Numerous studies and governmental regulations underscore the risks associated with advanced maternal age, which may lead to potential complications for both the mother and the child. Despite the increasing restrictions imposed, it is crucial to inform prospective parents that successful conception at an advanced age still does not preclude serious implications for the child’s health such as an elevated likelihood of DNMs. Consequently, the focus should extend beyond merely regulating access to ART; it should also encompass the provision of comprehensive information regarding potential risks and their implications for the family.

## CONCLUSIONS AND FUTURE PERSPECTIVES

**Fig. 2:**
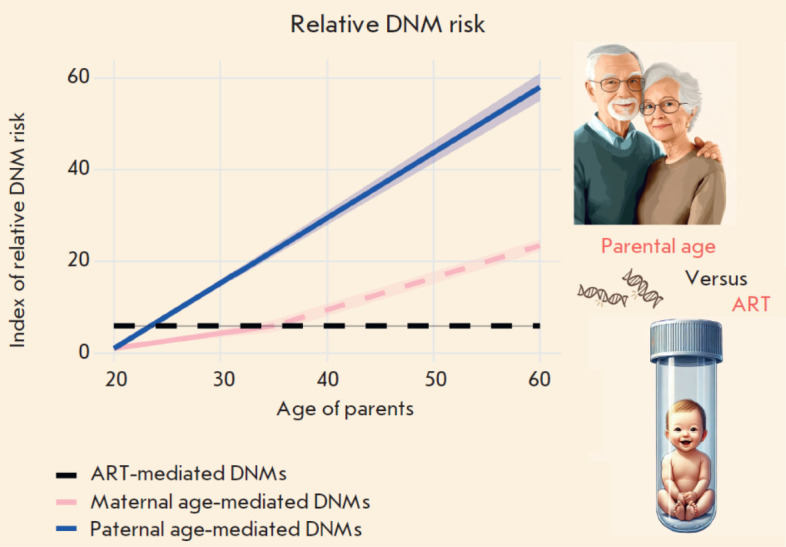
The impact of parental age and ART on the relative risk of *de novo *mutations (DNMs). The X axis represents parental age, and the Y axis shows the index of relative risk for DNMs, normalized to the minimal risk level observed at age 20. An increase in the index indicates an elevated likelihood of *de novo *mutations. The shaded areas surrounding the lines represent the range of potential risks, which is influenced by the variability of the coefficients used in calculations. The dashed segment of the maternal DNMs line beyond age 35 indicates data revealing a pronounced acceleration in risk, although this increase remains unverified [56, 68]. The following formulas were used for calculating the index: Δpaternal: The paternal age contribution to DNMs risk: Δpaternal = (Age – 20) × kpaternal, where kpaternal = 1.35–1.5 [41, 42, 43, 44, 45, 46, 47]. Δmaternal: The maternal age contribution to DNMs risk: For ages up to 35: Δmaternal = (Age – 20) × kmaternal_low, where kmaternal_low = 0.24–0.42 [41, 42, 43, 44, 45, 46, 47]. For ages above 35: Δmaternal = Δmaternal (35) + (Age – 35) × kmaternal_high, where Δmaternal (35) is the risk value at age 35, calculated using the first formula, and kmaternal_high = 0.61 [42]. The risk associated with ART was calculated as a fixed additional contribution that is added to the overall parental risk, regardless of age, and amounts to ~ 4.25 [56]

The impact of ART as relates to DNMs in humans remains a contentious issue; nevertheless, data show that aging, and paternal aging in particular, exerts the most consequential influence on the number of DNMs, alongside reproductive disorders linked to environmental factors (Fig. 2). However, the limitations that come with the small sample sizes of most studies and the predominant use of ART by couples experiencing infertility may obscure the direct impact of the technique as relates to DNMs. Moreover, many existing studies fail to account for stillbirths and pregnancy terminations, which complicates the evaluation of lethal DNMs. To address these limitations, research on model organisms such as cattle, as well as investigations into the mutations in ART-conceived children using various culture media and protocols, could provide valuable insights. This review primarily examined the presence of an association between DNMs and ART, which complicates the evaluation of lethal DNMs epigenetic changes, thus requiring further detailed exploration. Through the examination of these studies, we aimed to gain insights regarding implications for future generations and underscore the growing need to investigate the long-term consequences of ART.
